# Error detection and representation in the olivo-cerebellar system

**DOI:** 10.3389/fncir.2013.00001

**Published:** 2013-02-22

**Authors:** Masao Ito

**Affiliations:** Senior Advisor's Office, RIKEN Brain Science InstituteWako, Saitama, Japan

**Keywords:** adaptation, error, internal model, microcomplex, motor learning

## Abstract

Complex spikes generated in a cerebellar Purkinje cell via a climbing fiber have been assumed to encode errors in the performance of neuronal circuits involving Purkinje cells. To reexamine this notion in this review, I analyzed structures of motor control systems involving the cerebellum. A dichotomy was found between the two types of error: sensory and motor errors play roles in the feedforward and feedback control conditions, respectively. To substantiate this dichotomy, here in this article I reviewed recent data on neuronal connections and signal contents of climbing fibers in the vestibuloocular reflex (VOR), optokinetic eye movement response, saccade, hand reaching, cursor tracking, as well as some other cases of motor control. In our studies, various sources of sensory and motor errors were located in the neuronal pathways leading to the inferior olive. We noted that during the course of evolution, control system structures involving the cerebellum changed rather radically from the prototype seen in the flocculonodular lobe and vermis to that applicable to the cerebellar hemisphere. Nevertheless, the dichotomy between sensory and motor errors is maintained.

## Introduction

Since the early discussions held around 1970 (see Ito, 2002), it has generally been assumed that climbing fibers emerging from the inferior olive convey error signals, which play a teacher's role in the learning mechanism of the cerebellum. However, where and how such error signals are derived and encoded in climbing fiber activities remains unclarified. In particular, problems regarding the sensory vs. motor nature of error signals and forward vs. inverse internal models have been debated (for example, Kobayashi et al., 1998; Winkelman and Frens, 2006; Ebner and Pasalar, 2008). Here, I attempt to clarify the problems by reexamining the control system structures of the cerebellum on the basis of recent experimental data on neuronal connections and signal contents of climbing fiber discharges in various cases of motor control involving the olivo-cerebellar system.

## Adaptive control system model of the cerebellum

**Figure 1:**
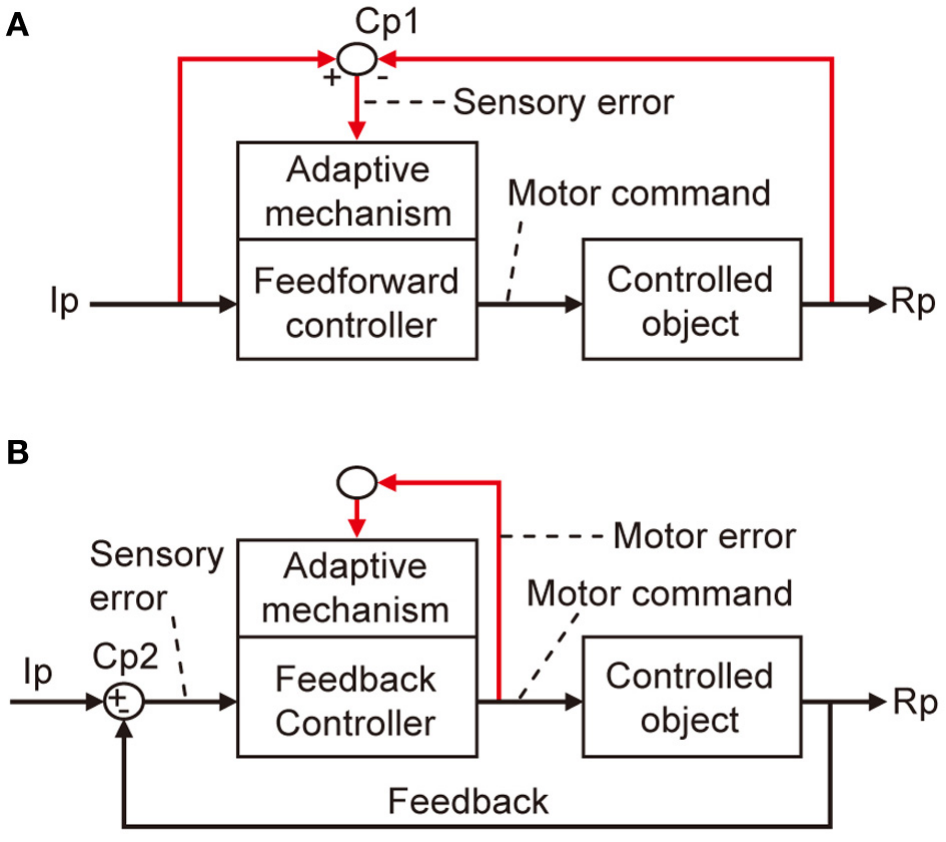
**Two types of adaptive control system. (A)** Without online feedback. **(B)** With online feedback. Pathways for computing error signals are indicated by red lines.

The errors that I am concerned with here are defined in terms of a basic scheme for adaptive control systems (Figures 1A,B). In this scheme, the controller converts instructions to motor commands, which in turn act on a controlled object that finally yields output responses to realize the instruction. An error is defined as the discrepancy between two lines of information, namely, instruction on the movement to be performed and information about the produced movement. Signals representing such an error then drive the adaptive mechanism attached to the controller.

There are two obvious ways to compute such an error as defined above. First, as shown in Figure 1A, when a control system performs feedforward control without an ongoing feedback, a comparator is required to compare between signals representing the desired movement and signals representing the performed movement received via respective pathways (red lines). These two lines of information are both represented in sensory coordinates as “kinematics” description of motion of the body or its parts in terms of position, velocity, acceleration, and direction. Errors derived from their comparison are also represented in sensory coordinates and are hence often called sensory errors.

In contrast, when a control system performs feedback control, the controller is driven by the difference between the desired and produced movements. In this situation, the controller itself acts as a comparator, as shown in Figure 1B. The sensory errors so derived at the inputs of the controller are readily converted by the controller to errors in motor commands, called motor errors. Motor errors are represented in motor-command coordinates in terms of “dynamics” description of motion such as force that causes motion. Kawato and his associates (Kawato et al., 1987; Kawato and Gomi, 1992a,b; Kawato, 1999) proposed an ingenious idea of feedback-error learning, that is, the motor errors derived by the primary motor cortex as a feedback controller play a crucial role in the learning mechanism of the cerebellum for arm movements (see below and Figure 3B). Based on these considerations, we here define sensory errors as detected by sensory systems and represented in sensory coordinates and motor errors as implied in motor commands and represented in motor command coordinates.

**Figure 2:**
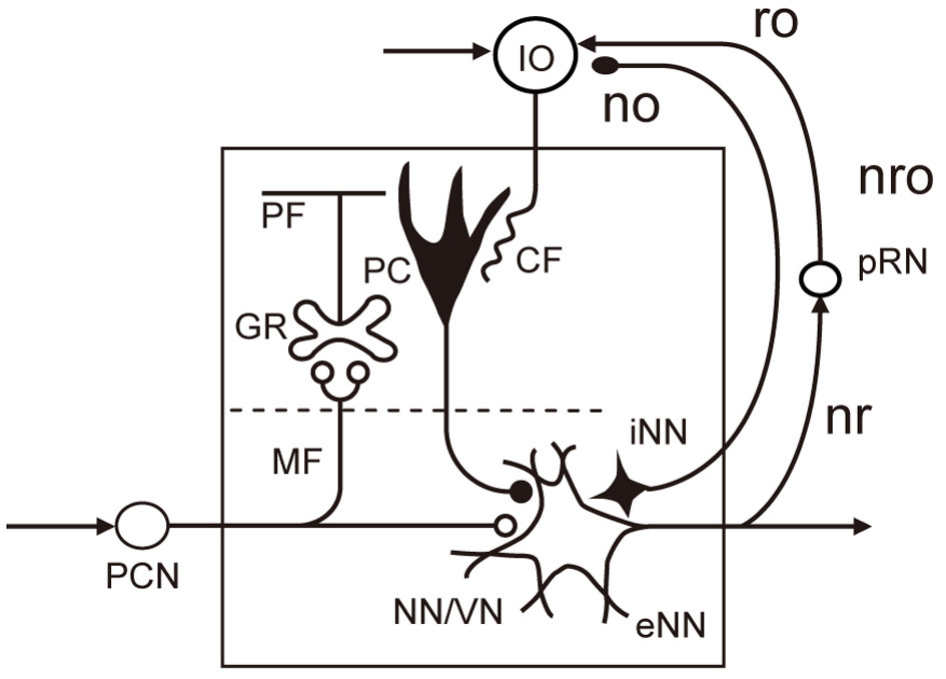
**Basic circuit structure of microcomplex.** Abbreviations: CF, climbing fiber; GR, granule cell; IO, inferior olive; MF, mossy fiber; NN/VN, nuclear neuron/vestibular nuclear neuron; no, nucleoolivary pathway; nro, nucleorubroolivary pathway; PC, Purkinje cell; PCN, precerebellar neuron; PF, parallel fiber; pRN, parvocellular red nucleus; eNN, excitatory nuclear neuron; iNN, inhibitory nuclear neuron; ro, rubroolivary pathway; nr, nucleorubral pathway Inhibitory neurons are in black (others are excitatory).

The microcomplex is the structural and functional unit module of neuronal circuits in the cerebellum derived from recent experimental studies (see Ito, 1984, 2006). It is a convenient biological concept corresponding to a block in control systems. As shown in Figure 2, each microcomplex consists of a microzone of the cerebellar cortex and associated cerebellar or vestibular nuclei. Each microcomplex receives major input signals via mossy fibers and converts them to an output of cerebellar or vestibular nuclear neurons. Each microcomplex also receives climbing fiber signals encoding errors for learning. (It also receives signals of beaded fibers for neuromodulation, but for simplicity, this will not be dealt with in this review). Climbing fibers originate from the inferior olive and pass the cerebellar cortex to supply strong excitatory synapses to Purkinje cells and other inhibitory interneurons. Collaterals of mossy fibers and climbing fibers also innervate cerebellar/vestibular nuclear neurons. Note that nuclear neurons, not Purkinje cells, are the output of the microcomplex.

The learning capability of a microcomplex is evident because microcomplex lesioning leads to the loss of adaptability in a relevant specific control function, as will be reviewed below. To discuss neural mechanisms of this learning capability in detail is beyond the scope of this review; hence, only two issues are mentioned here. One is that long-term potentiation/depression (LTP/LTD) as a neural substrate of memory is now located not only in the cerebellar cortex but also in the vestibular/cerebellar nucleus (see McElvain et al., 2010). Although synaptic plasticity is exhibited at many sites in the cerebellar tissues (D'Angelo et al., 1999; Hansel et al., 2001), LTD occurring at parallel fiber-Purkinje cell synapses conjunctively activated by climbing fibers has been considered to play a major role in fast adaptation, whereas LTP occurring at mossy fiber collateral-to-nuclear cell synapses accounts for slow adaptation (see below). These two types of adaptation are not parallel processes independent of each other, because cortical adaptation is required for initiation of nuclear adaptation (Kassardjian et al., 2005), as if memory trace shifts in time from the cortex to the nucleus (Okamoto et al., 2011). Another issue is that, although motor learning usually does not occur when LTD is impaired by pharmacological or genetic manipulation, the three types of mutant mouse generated by Schonewille et al. (2011) exhibit apparently normal motor learning even when LTD does not occur *in vitro* in cerebellar slices obtained from these mice. In another study, protein synthesis inhibitors readily blocked LTD induction *in vitro* (Karachot et al., 2001), but when injected into the cerebellar flocculus these inhibitors had virtually no effect on the fast optokinetic adaptation (see below), which has been related to LTD induction (Okamoto et al., 2011). This LTD-motor learning mismatch has been considered as being contradictory to the so-called Marr-Albus-Ito hypothesis, but I would like to point out that LTD is tested *in vitro* in slices, which function under fundamentally artificial conditions; slices are disconnected in electrical and chemical signaling from surrounding tissues and potential plasticity factors could be continuously washed out by perfusates. Moreover, to induce LTD in slices, artificial stimuli composed of electric pulses (repeated 300 times at 1 Hz) must be applied. It is possible that a disturbance could easily disrupt LTD induction *in vitro*, whereas LTD induction by natural stimuli under *in vivo* conditions might remain robust so that its blockade under similar conditions or perturbation could be relatively difficult. This possibility needs to be examined in future studies.

**Figure 3:**
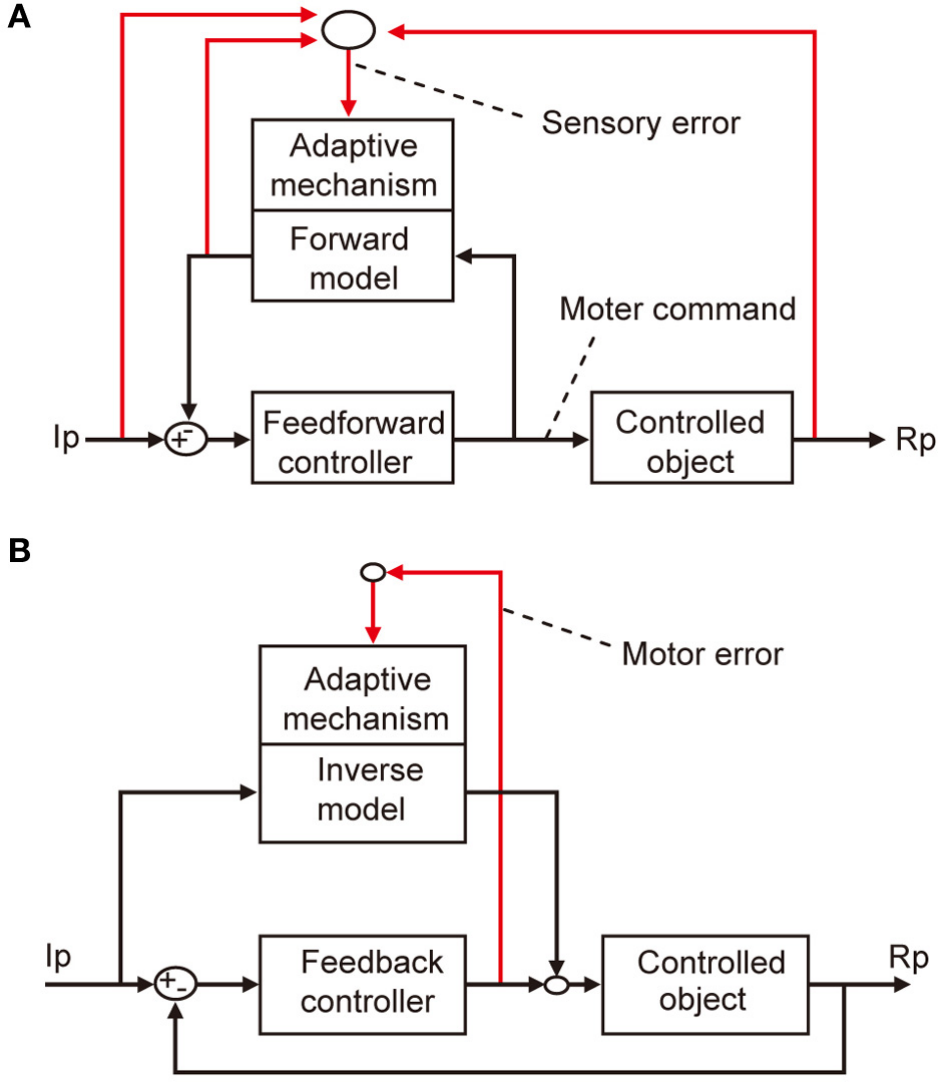
**Two types of internal model. (A)** Forward model linked to feedforward controller. **(B)** Inverse model linked to feedback controller.

The microcomplex provides a neural substrate of internal models incorporated in the cerebellar control system (Kawato et al., 1987; Wolpert et al., 1998). Two types of internal model of the controlled object have been defined. The “inverse” model has the inputs and outputs corresponding to the outputs and inputs of a controlled object, and can serve by itself as an adaptive feedforward controller (Figure 3A). The forward model, in contrast, has the input and output corresponding to the input and output of a controlled object, and simulates the performance of the controlled object in feedback control (Figure 3B).

## Vestibuloocular reflex (VOR)

**Figure 4:**
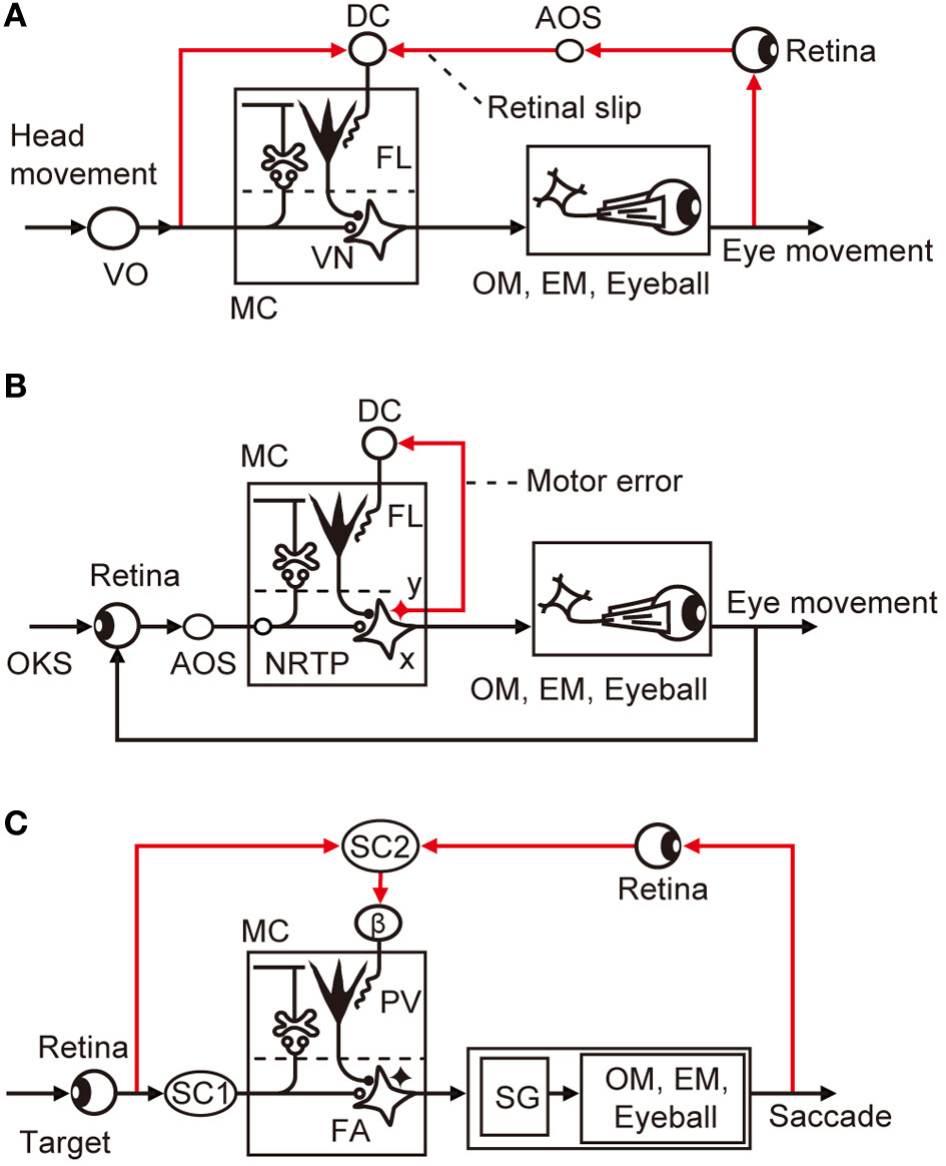
**Control system structure for three types of eye movement reflex. (A)** VOR. **(B)** OKR. **(C)** saccade. Additional abbreviations: β, β subnucleus of inferior olive; DC, dorsal cap; EM, extraocular muscle; FA, fastigial nucleus; NRTP, nucleus reticularis tegmenti pontis; OKS, optokinetic stimulus; OM, oculomotor neurons; SC, superior colliculus; SG, saccade generator; AOS, accessory optic system; FL, flocculus; VN, vestibular nuclear neuron; MC, microcomplex; VO, vestibular organ; PV, posterior vermis; x, vestbuloocular relay neuron; y, vestibular nuclear neuron working in parallel with x.

VOR has been explored as a model system of cerebellar control. As it is evoked by a head movement and causes a compensatory eye movement, VOR is a purely feedforward control lacking feedback (Figure 4A); hence, it should have a control system structure in which an adaptive mechanism is driven by sensory errors (Figure 1A). Note that VOR contains 14 component reflexes (Ezure and Graf, 1984) arising from six semicircular canals (three on each side) and four otolith organs (two on each side) and ending at different extraocular muscles (six on each side), but for simplicity, we focus on the horizontal canal-ocular reflex unless otherwise stated. When the head rotates ipsilaterally under illumination, the eyes rotate contralaterally to stabilize the retinal images of the external world. Here, the net discrepancy between the instruction given by head rotation via the vestibular organ and the information about the eye movements mediated by the retina represents sensory errors, which are called retinal slips.

Retinal slips can be manipulated by changing the relationship between head movements and movements of the visual environment using magnifying or minifying lenses, right-left converting prisms, or an inphase/outphase combination of head oscillation and screen oscillation. When an animal is continuously exposed to such manipulated retinal errors, the gain of VOR adaptively increases or decreases to minimize retinal slips. This paradigm causes the fast VOR adaptation that develops in 1 h and the slow adaptation that develops in 1 week (Kassardjian et al., 2005; Anzai et al., 2010). The fast VOR adaptation is mediated by the flocculus cortex, whereas the slow VOR adaptation is mediated by the vestibular nuclei.

Controller neurons for VOR are located in the vestibular nuclei; they receive excitatory inputs from vestibular afferents, which also project, directly or indirectly, to the flocculus as mossy fibers. VOR relay neurons also receive inhibitory innervation by Purkinje cells from a microzone in the flocculus (Sekimjak et al., 2003). Climbing fibers derived from the dorsal cap of the inferior olive project to floccular Purkinje cells. Dorsal cap neurons are activated by visual signals via the accessory optic system (AOS). The microcomplex so constructed constitutes an inverse model of the controlled object involving oculomotor neurons, extraocular muscles, and eyeballs, and is considered to serve as a feedforward adaptive controller for VOR (Figure 4A).

In Figure 4A, the dorsal cap is placed in the position for the comparator that computes retinal slips (compare with Figure 1A). It has been somewhat puzzling that floccular Purkinje cells receive climbing fiber signals during head rotation in darkness in the absence of vision to detect errors (Ghelarducci et al., 1975; Simpson et al., 2002). However, as seen in Figure 4A, vestibular signals represent an instruction on the extent to which the eyes should move to compensate for head movement, and are therefore important inputs to the comparator. How vestibular sensory signals reach the inferior olive is still unclear, but a likely candidate of relay is the nucleus prepositus hypoglossi, which passes major inhibitory inputs to the dorsal cap (De Zeeuw et al., 1993). The nucleus prepositus hypoglossi contains neurons sensitive to horizontal head rotation (Lannou et al., 1984; McFarland and Fuchs, 1992).

These observations support the view that the dorsal cap acts as the comparator for VOR. If VOR works as shown in Figure 4A, involvement of motor errors in VOR adaptation is unlikely because there is no way to generate feedback errors in this purely feedforward control system. However, caution is needed regarding the interpretation of the finding that, under illumination, VOR operates jointly with the optokinetic eye movement response (OKR), which is a feedback control and may therefore introduce motor errors (see below).

## OKR

OKR moves an eye to follow a relatively slowly moving visual environment. It is a feedback control system equipped with a visual feedback loop, and is a typical example in which a feedback controller generates motor errors. OKR exhibits an adaptive gain increase toward unity during prolonged sinusoidal oscillation of the visual environment around a stationary subject. Its short-term (fast) adaptation develops during a 1-h screen oscillation and diminishes throughout the subsequent 24 h. In contrast, long-term (slow) adaptation of OKR is established by repeated sessions of screen rotation for 7 days, and it persists for a week even after the flocculus is injected with locally acting lidocaine (Shutoh et al., 2006). Slow OKR adaptation is underlain by LTP in vestibular nerve-VOR relay neuron synapses. These lines of evidence suggest that the memory of fast OKR adaptation formed in the flocculus may subsequently induce the memory of slow adaptation in vestibular nuclear neurons (Okamoto et al., 2011).

In Figure 4B, we may assume that retinal slips are derived by comparing visually monitored eye movements with optokinetic stimuli at the inputs of the controller and are converted via the controller to motor errors. The motor errors so derived would drive the adaptive mechanism attached to the feedback controller as analyzed by Kawato and Gomi (1992b). Because there is no known recurrent collaterals of VOR relay neurons to the inferior olive, it is probable that a group of vestibular nuclear neurons, working in parallel to the VOR relay neurons, convey motor error signals to the dorsal cap (Figure 4A). A candidate for such neurons may also be found in the nucleus prepositus hypoglossi; it contains neurons not only sensitive to head velocity, as mentioned above, but also those sensitive to eye velocity (McFarland and Fuchs, 1992).

VOR and OKR share the same controller (microcomplex) and controlled object. The adaptive mechanism is also shared commonly so that VOR and OKR are simultaneously adapted even when exposed to adaptation separately. Indeed, sustained sinusoidal oscillation of a striped cylindrical screen around a stationary, alert pigmented rabbit for 4 h not only increased the OKR gain by 0.23, but also induced a simultaneous increase in the VOR gain by 0.18 (Nagao, 1989). Purkinje cell spikes recorded from the floccular areas related to horizontal eye movements (H-zone) normally exhibit modulation of simple spike discharges in phase with screen velocity and out of phase with turntable velocity. Sustained screen oscillation for 1 h enhanced the simple spike responses to not only the screen but also the turntable oscillation. These observations suggest that the adaptive mechanism of the controller is common to VOR and OKR.

Because of the overlap of neuronal circuits for VOR and OKR, it may be expected that the dorsal cap mediates both sensory and motor errors. However, it is generally difficult to isolate movement-related motor signals from stimulus-induced sensory signals because the stimulus causes movements that generate motor signals. Winkelman and Frens (2006) applied visual motion noise stimuli to a rabbit to break the tight relationship between instantaneous visual stimuli and eye movements. They found that climbing fiber signals contain motor signals two-fold the sensory signals. However, caution is needed in interpreting this finding because movement-related signals so detected may not always represent genuine motor errors (errors in motor commands); some of them may merely reflect visual perception of eye movements.

When evoked with a single moving dot pattern, climbing fiber signals are related to retinal slips. However, when Frens et al. (2001) applied two sets of patterns (one stationary and the other moving) in superposition, thereby generating two sets of differently moving retinal slips in superposition, the evoked climbing fiber discharges to floccular Purkinje cells were modulated by retinal slips generated by the moving dots, but not by the mixed retinal slips. Frens et al. (2001) interpreted this observation as negating the proposition that climbing fiber discharges represent retinal slips. Nevertheless, how rabbit AOS responds to such mixed retinal slips has not been clarified, and the possibility that two sets of retinal slips presented simultaneously interact non-linearly with each other in the visual pathway mediated by AOS should be examined.

## Saccadic eye movement

A saccade is a quick, simultaneous movements of both eyes in the same direction to catch a visual target by small foveal areas of the retinas for high-acuity vision. Because of the very rapid (ballistic) eye movements in a saccade, it is not possible to control moving eyes by ongoing visual feedback; it is a purely feedforward control to which the sensory-error-learning scheme in Figure 1A should typically apply. Errors in a saccade are detected by comparison between the shift of the target spot in the retina that provokes a saccade and the shift of the eye position made by the saccade, both being projected to the map of the superior colliculus. The saccadic adaptation can be induced by repeatedly shifting the target while a saccadic movement is under way (for example, from 15° to 10° or 20°, the resultant eye position ending 5° long or short of the target). The saccade to the initial target position is followed by a corrective saccade to hit the shifted target position. After repeated trials, the eyes become able to catch the shifted position by only one saccade. This paradigm provides a favorable opportunity to explore the neuronal mechanism of error representation in climbing fibers (Soetedjo et al., 2008; Kojima et al., 2010).

Lesions in the posterior vermis permanently abolished this saccadic adaptation (McLaughlin, 1967). In most Purkinje cells tested in this area by Soetedjo et al. (2008), the probability of complex spike firing increases in the “error interval” between the primary and corrective saccades. In most Purkinje cells, complex spikes occur around 100 ms after the error onset. The probability of complex spike occurrence depends on both error direction and size. These observations are in good agreement with the concept that complex spikes encode sensory errors (although some different observations and interpretations were reported by Catz et al., 2005). Kojima et al. (2007) located the pathway that conveys such errors in saccadic adaptation in the monkey midbrain tegmentum. Weak electrical stimulation of this pathway at ~200 ms after a saccade in one horizontal direction produces changes in saccade gain, similar to changes induced by adaptation to real visual errors. This pathway appears to emerge from the superior colliculus and projects to subnucleus β of the medial accessory nucleus of the inferior olive, which in turn projects to the posterior vermis (Yamada and Noda, 1987; Kyuhou and Matsuzaki, 1991).

The microcomplex for control of saccade involves the posterior vermal cortex and caudal portion of the fastigial nucleus (Robinson et al., 1993). In depicting the entire control system structure for saccades in Figure 4C, the brainstem saccade generator circuit (Ramat et al., 2007) is placed within the control object that also includes the oculomotor system with eyeballs. The subnucleus β receives a strong input from the intermediate layer of the superior colliculus (Huerta and Harting, 1984), which contains neurons that discharge maximally for visual stimuli falling on a particular area of the contralateral hemifield (for review, see Sparks and Hartwich-Young, 1989). One may assume that these neurons could mediate the visual information of the target before and after saccades, from which error signals are produced at the β nucleus. The intermediate layer of the superior colliculus also contains many neurons that discharge prior to a saccade. This motor activity, however, seems irrelevant to climbing fibers because no complex spikes discharge to the dorsal vermis with a clear relationship to eye movements in the saccade (Soetedjo et al., 2008).

## Hand reach and cursor tracking

In the hand-reach paradigm adopted by Kitazawa et al. (1998), the monkey saw its hand and fingers and the target before and after completion of the movement, but the movement itself was performed without visual feedback. In this case, Purkinje cells in cerebellar lobules HIV–HVI exhibited multiply timed climbing fiber responses at three different stages of the movement (first, second, and third responses). The third response occurred at the end point of the movement, apparently representing visually perceived deviations between the target and the reaching finger's end position. However, the first and second Purkinje cell responses appeared too early to be interpreted similarly.

On the other hand, in the visually guided wrist tracking movement adopted by Mano et al. (1986), a monkey followed a moving target with a cursor in the screen, which was driven with a handle moved by flexion or extension of the wrist. In this visually guided wrist tracking, Purkinje cells in the cerebellar hemisphere (lobules IV–VI) exhibited complex spikes related to movements, presumably reflecting motor errors. In another experiment by Wang et al. (1987), a monkey moved a manipulandum, the position of which is represented by a cursor on the screen. The monkey was trained to move the cursor from the start box to the target box, and the target box could be repositioned during movement. In this feedback control situation, climbing fiber discharges from Purkinje cells increased at various times of movement, apparently reflecting motor errors.

**Figure 5:**
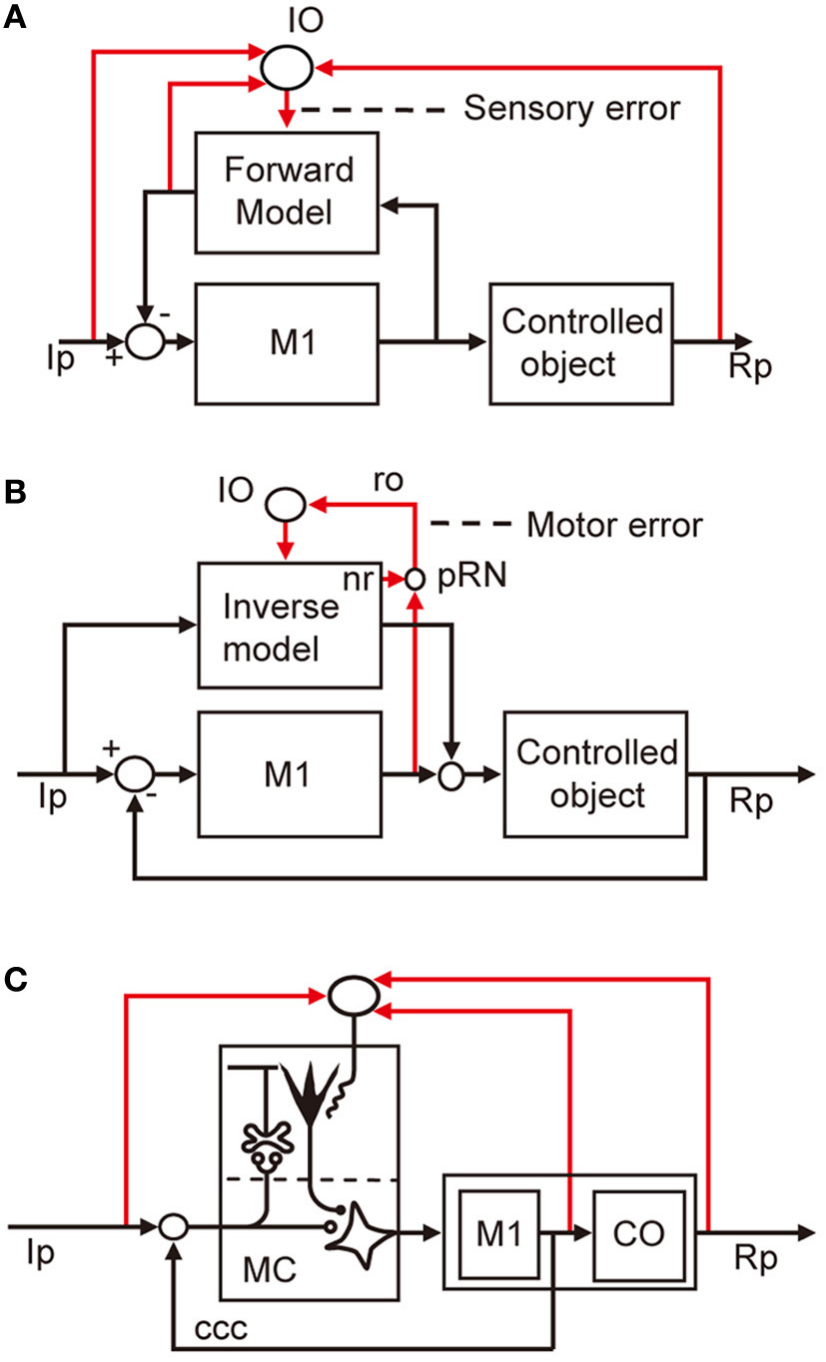
**Models for voluntary motor control. (A)** Forward-model-based control of arm movement by primary motor cortex. **(B)** Inverse-model-based control. **(C)** Application of reflex control scheme. Additional abbreviations: ccc, cerebrocerebellar communication loop. CO, control object including segmental circuit and skeletomuscular system; MC, microcomplex, M1, primary motor cortex; IO, inferior olive; Ip, instructed performance; Rp, realized performance; ro, rubroolivary pathway; pRN, parvocellular red nucleus; nr, nucleorubral pathway.

Control system models incorporating forward and inverse models have been proposed for voluntary arm movements, since the pioneering works by Kawato et al. (1987). In these models, the primary motor cortex is considered to play the role of the controller, whereas a microcomplex functions as a forward or inverse model of the controlled object that involves segmental circuits and skeletomuscular systems of the arm. In Figure 5A, a microcomplex provides a forward model, which forms a circular connection with the primary motor cortex, corresponding to the classic cerebrocerebellar communication loop. The internal feedback through the forward model is assumed to enable the motor cortex to predict consequences of the movement to be performed before the actual performance. In the situation in which the primary motor cortex operates in the feedforward manner, sensory errors should be processed by comparing the instruction, consequence, and prediction of movements, and passed to the forward model via the inferior olive (Figure 5A). The above-mentioned results of Kitazawa et al. (1998) can be explained on the basis of such a forward model. By contrast, in Figure 5B, a microcomplex provides an inverse model operating in parallel with the primary motor cortex. When the primary motor cortex functions as a feedback controller, it derives motor error signals, and passes them to the inverse model via the inferior olive. This inverse-model-based control explains well the above-mentioned experimental observations by Mano et al. (1986) and Wang et al. (1987). These model-based control systems will be considered later in comparison with the model systems postulated above for VOR, OKR, and saccade.

## Other cases of cerebellar control

In addition to VOR, saccade, and hand reaching examined above, the classical eye blink conditioning (see Thompson, 1988) and nociceptive withdrawal reflex (see Apps and Garwicz, 2005) are typical examples of a control system lacking feedback and receiving sensory errors to the inferior olive. The ocular following response (OFR) is an eye-movement reflex elicited by brief, unexpected movements of a visual scene such as 100 ms ramp changes (Kawano and Miles, 1986; Miles and Kawano, 1986). OFR may appear to operate by feedback, but in its early phase it is a feedforward mechanism because of its long loop time (0.1 s, Smith et al., 1969; Khan and Franks, 2003) for visual information processing across the retina. This mixture of feedforward and feedback control in OFR may explain the finding that climbing fibers to Purkinje cells in the ventral paraflocculus represent both sensory and motor errors (Kobayashi et al., 1998).

On the other hand, when feedback control is performed by OKR or cursor trackings, as described above, climbing fiber discharges represent motor errors. Smooth pursuit eye movements to follow a moving spot should be a feedback control, but the feedback is non-functional during the initial 100 m after the onset of movement of a target; here, eyes are driven in the feedforward manner because of the retinal delay (Smith et al., 1969). To manipulate motor errors independently of sensory errors, Yamamoto et al. (2007) trained monkeys to flex or extend the elbow by 45° in 400 ms under resistive and assistive force fields but without altering sensory measures of the movement such as velocity profiles. Unfortunately, the relationship of climbing fiber signals with muscle activities has not been reported.

It now appears that learning in a microcomplex is driven by either sensory or motor errors depending on the situation of whether a motor system performs the feedforward (or offline feedback) or feedback control. Both sensory and motor errors could access the inferior olive, but their representation would change depending on the circumstantial conditions.

## Sources of errors

From the studies reviewed above, sensory errors are determined to be derived at various preolivary structures and finally converted to climbing fiber signals at the inferior olive. Typically, in VOR, retinal slip signals are derived by comparison between the vestibular and visual signals at the dorsal cap of the inferior olive (Figure 4A). In OKR, on the other hand, retinal slip signals are derived by comparison between the instructed and produced eye movements at the retina from which the retinal slip signals pass via AOS to the dorsal cap. For saccades, the superior colliculus detects errors and sends them to the β subnucleus of the inferior olive (Figure 4C).

On the other hand, when a feedback controller derives motor errors as feedback errors (Figure 1B), how are these motor errors conveyed to the inferior olive? A likely possibility is that involved in the classic dentatorubroolivary triangle (nro in Figure 2). There, large excitatory nuclear neurons mediating the outputs of a microcomplex project to certain midbrain structures including the parvocellular red nucleus, the nucleus of Cajal, and the nucleus of Darkschwitsch (Saint-Cyr and Courville, 2004; see also Glickstein et al., 2011). Neurons of these nuclei in turn project excitatory synapses to the inferior olive. In addition, the smaller inhibitory neurons in the cerebellar nuclei receive excitatory and inhibitory inputs in parallel with the larger excitatory nuclear neurons, and in turn send inhibitory connections to the inferior olive. A likely possibility (which is yet to be explored) is that these excitatory and inhibitory pathways mediate motor error signals to the inferior olive. However, a reservation must be made because this possibility does not fit the model for the internal model-based control of voluntary movement (see below).

For either sensory or motor errors, the inferior olive is the final station of the pathways along which errors are computed. The actual role of inferior olive neurons may be to recode the high-frequency information carried by their synaptic inputs into stochastic, low-rate discharges in their climbing fiber outputs (Schweighofer et al., 2004). Inferior olive neurons are mutually connected by gap-junction-mediated electrical synapses, and activation or inhibition of these synapses determines whether climbing fiber activity is rhythmic, random, or synchronous (Kitazawa and Wolpert, 2005), switching the efficacy of error signals in learning.

## Evolutionary gap

We have seen above that current models of the cerebellar control system have a gap between the phylogenetically old flocculonodular lobe and vermis (Figure 4) and the phylogenetically newer intermediate part of the cerebellar hemisphere (Figure 5). In particular, the microcomplex involved acts as a controller in the former, whereas it acts in the latter as an internal model linked to the controller in the primary motor cortex. Such a drastic change may not be impossible when one considers the remarkable evolutionary growth of the cerebellum in mammals; from the flocculonodular lobe/vermis linked with the brainstem, dominance shifts to the cerebellar hemisphere (intermediate and lateral parts) linked with the cerebral neocortex. The microcomplexes appear to be half-buried in the brainstem circuits in the former, whereas these appear to be free to be incorporated as internal models in the newly evolved controllers in the neocortex.

The internal-model-based control explains well the events in the cerebellum during voluntary arm movements. However, when these models are adopted, one must find a functional role of the nucleorubroolivary pathway other than conveying motor errors. It has been suggested that motor errors generated by the primary motor cortex are passed to the parvocellular red nucleus to join the nucleorubroolivary pathway (Kawato et al., 1987). However, as yet, we have no idea about the possible function of the nucleorubral part of the pathway (Figure 2).

We also considered applying the adaptive control model for the flocculonodular lobe and vermis to the cerebellar hemisphere. We designated the microcomplex including the intermediate part of the cerebellar hemisphere and interpositus nucleus as the controller and the primary motor cortex as part of the controlled object (Figure 5C). To adopt this model, however, we need to clarify the role of the cerebrocerebellar communication loop linking the primary motor cortex with the cerebellar hemisphere. Obviously, more data on neuronal connections and signal contents of climbing fibers are required to fill the gap we face in demonstrating consistent neural designs of entire cerebellar control systems.

### Conflict of interest statement

The author declares that the research was conducted in the absence of any commercial or financial relationships that could be construed as a potential conflict of interest.

## References

[B1] Anzai M. Kitazawa H. Nagao S. (2010). Effects of reversible pharmacological shutdown of cerebellar flocculus on the memory of long-term horizontal vestibulo-ocular reflex adaptation in monkeys. Neurosci. Res. 68, 191–198 10.1016/j.neures.2010.07.2038 20674618

[B2] Apps R. Garwicz M. (2005). Anatomical and physiological foundations of cerebellar information processing. Nat. Rev. Neurosci. 6, 297–311 10.1038/nrn1646 15803161

[B3] Catz N. Dicke P. W. Their P. (2005). Cerebellar complex spike firing is suitable to induce as well as to stabilize motor learning. Curr. Biol. 15, 2179–2189 10.1016/j.cub.2005.11.037 16360681

[B4] D'Angelo E. Rossi P. Armano S. Taglietti V. (1999). Evidence for NMDA and mGlu receptordependent long-term potentiation of mossy fiber-granule cell transmission in rat cerebellum. J. Neurophysiol. 81, 277–287 9914288 10.1152/jn.1999.81.1.277

[B5] De Zeeuw C. I. Wentzel P. Mugnaini E. (1993). Fine structure of the dorsal cap of the inferior olive and its GABAergic and non-GABAergic input from the nucleus prepositus hypoglossi in rat and rabbit. J. Comp. Neurol. 327, 63–82 10.1002/cne.903270106 7679420

[B6] Ebner T. J. Pasalar S. (2008). Cerebellum predicts the future motor state. Cerebellum 7, 583–588 10.1007/s12311-008-0059-3 18850258 PMC2754147

[B7] Ezure K. Graf W. (1984). A quantitative analysis of the spatial organization of the vestibulo-ocular reflexes in lateral- and frontal-eyed animals—II. Neuronal networks underlying vestibulo-oculomotor coordination. Neuroscience 12, 95–109 10.1016/0306-4522(84)90141-6 6611518

[B8] Frens M. A. Mathoera A. L. van der Steen J. (2001). Floccular complex spike response to transparent retinal slip. Neuron 30, 795–801 11430812 10.1016/s0896-6273(01)00321-x

[B9] Ghelarducci B. Ito M. Yagi N. (1975). Impulse discharges from floccular Purkinje cells of alert rabbits during visual stimulation combined with horizontal head rotation. Brain Res. 87, 66–72 1078987 10.1016/0006-8993(75)90780-5

[B10] Glickstein M. Sultan F. Voogd J. (2011). Functional localization in the cerebellum. Cortex 47, 59–80 10.1016/j.cortex.2009.09.001 19833328

[B12] Hansel C. Linden D. J. D'Angelo E. (2001). Beyond parallel fiber LTD: the diversity of synaptic and non-synaptic plasticity in the cerebellum. Nat. Neurosci. 4, 467–475 10.1038/87419 11319554

[B13] Huerta M. F. Harting J. K. (1984). Comparative Neurology of the Optic Tectum, ed Vanegas H. (New York, NY: Plenum Press), 687–773

[B14] Ito M. (1984). The Cerebellum and Neural Control. New York, NY: Raven Press

[B15] Ito M. (2002). Historical review of the significance of the cerebellum and the role of purkinje cells in motor learning. Ann. N.Y. Acad. Sci. 978, 273–288 10.1111/j.1749-6632.2002.tb07574.x 12582060

[B16] Ito M. (2006). Cerebellar circuitry as a neuronal machine. Prog. Neurobiol. 78, 272–303 10.1016/j.pneurobio.2006.02.006 16759785

[B17] Karachot L. Shirai Y. Vigot R. Yamamori T. Ito M. (2001). Induction of long-term depression in cerebellar Purkinje cells requires a quickly turned over protein. J. Neurophysiol. 86, 280–289 11431509 10.1152/jn.2001.86.1.280

[B18] Kassardjian C. D. Tan Y.-F. Chung J.-Y. Heskin R. Peterson M. J. Broussard D. M. (2005). The site of amemory shifts with consolidation. J. Neurosci. 25, 7979–7985 10.1523/JNEUROSCI.2215-05.2005 16135754 PMC6725450

[B19] Kawano K. Miles F. A. (1986). Short-latency ocular following responses of monkey. II. Dependence on a prior saccadic eye movement. J. Neurophysiol. 56, 1355–1380 3794773 10.1152/jn.1986.56.5.1355

[B20] Kawato M. (1999). Internal models for motor control and trajectory planning. Curr. Opin. Neurobiol. 9, 718–727 10.1016/S0959-4388(99)00028-8 10607637

[B21] Kawato M. Furukawa K. Suzuki R. (1987). A hierarchical neural-network model for control and learning of voluntary movement. Biol. Cybern. 57, 169–185 3676355 10.1007/BF00364149

[B22] Kawato M. Gomi H. (1992a). A computational model of four regions of the cerebellum based on feedback-error learning. Biol. Cybern. 68, 95–103 1486143 10.1007/BF00201431

[B11] Kawato M. Gomi H. (1992b). The cerebellum and VOR/OKR learning models. Trends Neurosci. 15, 445–453 10.1016/0166-2236(92)90008-V 1281352

[B23] Khan M. A. Franks I. M. (2003). Online versus offline processing of visual feedback in the production of component submovements. J. Mot. Behav. 35, 286–295 10.1080/00222890309602141 12873843

[B24] Kitazawa S. Kimura T. Yin P. B. (1998). Cerebellar complex spikes encode both destinations and errors in arm movements. Nature 392, 494–497 10.1038/33141 9548253

[B25] Kitazawa S. Wolpert D. M. (2005). Rhythmicity, randomness and synchrony in climbing fiber signals. Trends Neurosci. 28, 611–619 10.1016/j.tins.2005.09.004 16182386

[B26] Kobayashi Y. Kawano K. Takemura A. Inoue Y. Kitama T. Gomi H. (1998). Temporal firing patterns of Purkinje cells in cerebellar ventral paraflocculus during ocular following responses in monkeys. II. Complex spikes. J. Neurophysiol. 80, 832–848 9705472 10.1152/jn.1998.80.2.832

[B27] Kojima Y. Soetedjo R. Fuchs A. F. (2010). Changes in simple spike activity of some purkinje cells in the oculomotor vermis during saccade adaptation are appropriate to participate in motor learning. J. Neurosci. 30, 3715–3727 10.1523/JNEUROSCI.4953-09.2010 20220005 PMC2864307

[B28] Kojima Y. Yoshida K. Iwamoto Y. (2007). Microstimulation of the midbrain tegmentum creates learning signals for saccade adaptation. J. Neurosci. 27, 3759–3767 10.1523/JNEUROSCI.4958-06.2007 17409240 PMC6672395

[B29] Kyuhou S. Matsuzaki R. (1991). Topographical organization of the tecto-olivo- cerebellar projection in the cat. Neuroscience 41, 227–241 1711650 10.1016/0306-4522(91)90212-7

[B30] Lannou J. Cazin L. Precht W. Le Taillanter M. (1984). Responses of prepositus hypoglossi neurons to optokinetic and vestibular stimulations in the rat. Brain Res. 301, 39–45 10.1016/0006-8993(84)90400-1 6329446

[B31] Mano N. Kanazawa I. Yamamoto K. (1986). Complex-spike activity of cerebellar Purkinje cells related to wrist tracking movement in monkey. J. Neurophysiol. 56, 137–158 3746392 10.1152/jn.1986.56.1.137

[B32] McElvain L. E. Bagnall M. W. Sakatos A. du Lac S. (2010). Bidirectional plasticity gated by hyperpolarization controls the gain of postsynaptic firing responses at central vestibular nerve synapses. Neuron 68, 763–775 10.1016/j.neuron.2010.09.025 21092864 PMC3189222

[B33] McFarland J. L. Fuchs A. F. (1992). Discharge patterns in nucleus prepositus hypoglossi and adjacent medial vestibular nucleus during horizontal eye movement in behaving macaques. J. Neurophysiol. 68, 319–332 1517825 10.1152/jn.1992.68.1.319

[B34] McLaughlin S. C. (1967). Parametric adjustment in saccadic eye movements. Percept. Psychophys. 2, 359–362

[B35] Miles F. A. Kawano K. (1986). Short-latency ocular following responses of monkey. III. Plasticity. J. Neurophysiol. 56, 1381–1396 3794774 10.1152/jn.1986.56.5.1381

[B36] Nagao S. (1989). Role of cerebellar flocculus in adaptive interaction between optokinetic eye movement response and vestibulo-ocular reflex in pigmented rabbits. Exp. Brain Res. 77, 541–551 2806446 10.1007/BF00249607

[B37] Okamoto T. Endo S. Shirao T. Nagao S. (2011). Role of cerebellar cortical protein synthesis in transfer of memory trace of cerebellum-dependent motor learning. J. Neurosci. 31, 8958–8966 10.1523/JNEUROSCI.1151-11.2011 21677179 PMC6622939

[B38] Ramat S. Leigh R. J. Zee D. S. Optican L. M. (2007). What clinical disorders tell us about the neural control of saccadic eye movements. Brain 130, 10–35 10.1093/brain/awl309 17121745

[B39] Robinson F. R. Straube A. Fuchs A. F. (1993). Role of the caudal fastigial nucleus in saccade generation. II. Effects of muscimol inactivation. J. Neurophysiol. 70, 1741–1758 8294950 10.1152/jn.1993.70.5.1741

[B40] Saint-Cyr J. A. Courville J. (2004). Sources of descending afferents to the inferior olive from the upper brain stem in the cat as revealed by the retrograde transport of horseradish peroxidase. J. Comp. Neurol. 198, 567–581 10.1002/cne.901980403 7251931

[B41] Schonewille M. Gao Z. Boele H.-J. Veloz M. F. V. Amerika W. E. Simek A. A. M. (2011). Reevaluating the role of LTD in cerebellar motor learning. Neuron 70, 43–50 10.1016/j.neuron.2011.02.044 21482355 PMC3104468

[B42] Schweighofer N. Doya K. Fukai H. Chiron J. V. Furukawa T. Kawato M. (2004). Chaos may enhance information transmission in the inferior olive. Proc. Natl. Acad. Sci. U.S.A. 101, 4655–4660 10.1073/pnas.0305966101 15070773 PMC384802

[B43] Sekimjak C. Vissel B. Bollinger J. Faulstich M. du Lac S. (2003). Purkinje cell synapses target physiologically unique brainstem neurons. J. Neurosci. 23, 6392–6398 12867525 10.1523/JNEUROSCI.23-15-06392.2003 PMC6740533

[B44] Shutoh F. Ohki M. Kitazwa H. Itohara S. Nagao S. (2006). Memory trace of motor learning shifts transsynaptically from cerebellar cortex to nuclei for consolidation. Neuroscience 139, 767–777 10.1016/j.neuroscience.2005.12.035 16458438

[B45] Simpson J. I. Belton T. Suh M. Winkelman B. (2002). Complex spike activity in the flocculus signals more than the eye can see. Ann. N.Y. Acad. Sci. 978, 232–236 10.1111/j.1749-6632.2002.tb07570.x 12582056

[B46] Smith K. U. Putz V. Molitor K. (1969). Eye movement-retina delayed feedback. Science 166, 1542–1544 17695081 10.1126/science.166.3912.1542

[B47] Soetedjo R. Kojima Y. Fuchs A. F. (2008). Complex spike activity in the oculomotor vermis of the cerebellum: a vectorial error signal for saccade motor learning? J. Neurophysiol. 100, 1949–1966 10.1152/jn.90526.2008 18650308 PMC2576224

[B48] Sparks D. L. Hartwich-Young R. (1989). The deep layers of the superior colliculus. Rev. Oculomot. Res. 3, 213–255 2486324

[B49] Thompson R. F. (1988). The neural basis of basic associative learning of discrete behavioral responses. Trends Neurosci. 11, 152–155 10.1016/0166-2236(88)90141-5 2469183

[B50] Wang J.-J. Kim J. H. Ebner T. J. (1987). Climbing fiber afferent modulation during a visually guided, multi-joint arm movement in the monkey. Brain Res. 410, 323–329 10.1016/0006-8993(87)90331-3 3594241

[B51] Winkelman B. H. Frens M. A. (2006). Motor coding in floccular climbing fibers. J. Neurophysiol. 95, 2342–2351 10.1152/jn.01191.2005 16354726

[B52] Wolpert D. M. Miall R. C. Kawato M. (1998). Internal models in the cerebellum. Trends Cogn. Sci. 2, 338–347 21227230 10.1016/s1364-6613(98)01221-2

[B53] Yamada J. Noda H. (1987). Afferent and efferent connections of the oculomotor cerebellar vermis in the macaque monkey. J. Comp. Neurol. 265, 224–241 10.1002/cne.902650207 3320110

[B54] Yamamoto K. Kawato M. Kotosaka S. Kitazawa S. (2007). Encoding of movement dynamics by Purkinje cell simple spike activity during fast arm movements under resistive and assistive force fields. J. Neurophysiol. 97, 1588–1599 10.1152/jn.00206.2006 17079350

